# Detection of New *Leptospira* Genotypes Infecting Symptomatic Dogs: Is a New Vaccine Formulation Needed?

**DOI:** 10.3390/pathogens9060484

**Published:** 2020-06-18

**Authors:** Cristina Bertasio, Maria Beatrice Boniotti, Laura Lucchese, Letizia Ceglie, Laura Bellinati, Matteo Mazzucato, Tommaso Furlanello, Mario D’Incau, Alda Natale

**Affiliations:** 1 National Reference Centre for Animal Leptospirosis, Istituto Zooprofilattico Sperimentale della Lombardia ed Emilia Romagna “Bruno Ubertini”, 25121 Brescia, Italy; mariabeatrice.boniotti@izsler.it (M.B.B.); mario.dincau@izsler.it (M.D.); 2 Istituto Zooprofilattico Sperimentale delle Venezie, Viale dell’Università, 35020 Legnaro, Italy; llucchese@izsvenezie.it (L.L.); lceglie@izsvenezie.it (L.C.); lbellinati@izsvenezie.it (L.B.); mmazzucato@izsvenezie.it (M.M.); anatale@izsvenezie.it (A.N.); 3 “San Marco” Veterinary Clinica and Laboratory, Via dell’ Industria, 35030 Veggiano, Italy; tf@sanmarcovet.it

**Keywords:** leptospirosis, dog, real-time PCR, genotyping, epidemiology, multilocus sequence typing, multiple loci variable-number tandem repeat analysis

## Abstract

Leptospirosis in dogs has been largely described worldwide, and epidemiological studies have been mainly based on serological data. This study aims to detect and genotype leptospires affecting symptomatic dogs in Northeast Italy between 2013 and 2019. Overall, 1631 dogs were tested using real-time PCR, and leptospires from 193 dogs were subjected to Multilocus Sequence Typing and a Multiple Loci Variable-number Tandem Repeat Analysis. Leptospires were successfully isolated from 15 symptomatic dogs. Six distinct Sequence Types (STs) were found for 135 leptospires, with 3 STs characterizing *Leptospira interrogans* (ST17, ST198 and ST24), 2 STs characterizing *Leptospira kirschneri* (ST117 and ST289) and 1 ST characterizing *Leptospira borgpetersenii* (ST155), revealing the circulation of the serogroups Icterohaemorrhagiae, Australis, Sejroe and Pomona. The Multiple Loci Variable-number Tandem Repeat Analysis of 17 samples did not result in any additional discrimination. Genotypes were compared with those of strains present in the historical internal database, and possible transmission chains were identified from rat, mouse, hedgehog and pig. This work highlights the importance of molecular methods in revealing and identifying circulating *Leptospira* strains, and it also encourages the evaluation of the ability of commercially available vaccines to reduce the disease burden among dogs.

## 1. Introduction

Leptospirosis is a widespread zoonotic disease caused by infection with a pathogenic species of the genus Leptospira [1]. It is a worldwide public health and veterinary problem involving many domestic and wild animal species. Dogs have been known to be hosts of pathogenic leptospires since 1931, when Klarenbeek and Schuffner first isolated leptospires from the urine of a dog affected by nephritis [2]. Dogs are highly susceptible to infection, and they act as a sentinel species for the environmental risk to humans because of their high level of environmental exposure to pathogenic leptospires. Canine leptospirosis has been largely described worldwide [1,2,3], and its seroprevalence varies in accordance with geographic location, as follows: 1.8% in Australia [4], 7.3% in China [5], 9.9% in Brazil [6], 14.63% in Iran [7], 17.1% in the USA [8], 25.8% in Spain [9] and 71.1% in India [10]. In Italy, a 2002 survey conducted on kenneled dogs reported a seroprevalence of 29.4% [11], and national data collected in 2010–2011, involving more than 3000 dogs, described a seropositivity of 29.9% [12]. Nevertheless, these studies were not standardized, and some were partially biased by vaccine responses, the panel of serovars adopted for the Micro Agglutination Test (MAT) and sample collection, which was based on clinical suspects. In fact, a recent survey conducted in Italy on both ill and healthy dogs reported a lower seroprevalence of 8% [13].

Canine leptospirosis has mainly been associated with serovars Canicola and Icterohaemorrhagiae, two serogroups included within the *Leptospira interrogans* species. In Europe, protective vaccines for dogs against these serovars have been available for ~60 years [14]; however, recently, leptospirosis’ epidemiological situation in dogs has changed, and clinical syndromes have been described in association with serovars not included in the traditional vaccines [1,11,15,16]. Because Grippotyphosa and Bratislava have emerged as major causes of canine leptospirosis in Europe [12,17,18,19], new canine *Leptospira* vaccines containing antigens from up to four different serogroups, Canicola, Icterohaemorrhagiae, Australis and Grippotyphosa, have been introduced in the area [20,21]. In particular, trivalent (serogroups Canicola, Icterohaemorrhagiae and Grippothyphosa) and tetravalent (serogroups Canicola, Icterohaemorrhagiae, Grippothyphosa and Australis) vaccines have been licensed in European countries.

To date, epidemiological studies have been mainly based on serological diagnoses, particularly MAT, which is based on determining the ability of serial dilutions of the tested serum to agglutinate live leptospiral serovars in vitro. Agglutination is assessed by darkfield microscopy and suggests exposure to a serovar belonging to the corresponding serogroup (but not necessarily to the serovar tested) [22]. It is based on the specific antigenic determinants related to the structural heterogeneity of the lipopolysaccharide, and it is considered the diagnostic test of choice in dogs suspected of having leptospirosis [16,23]. Despite the widespread use of MAT to diagnose leptospirosis in dogs, this assay has limitations linked to its indirect diagnostic nature, such as possible false-negative results for initial infections, cross-reactions and paradoxical reactions early during the course of the disease [22,24,25], the variability of the selected antigenic panel and the subjective interpretation of the results. Furthermore, it generally does not discriminate between vaccinated and infected, increasing the difficulty of interpreting canine tests [26]. In addition, making direct comparisons between different studies is complicated by the variability in cut-off MAT titers used.

Several direct molecular assays, such as real-time PCR targeting various leptospiral genes, have been developed to support serological methods [27,28,29,30,31,32]. The diagnostic performances of different PCR assays are not equivalent [33], but they have been very useful in confirming diagnoses at the early stages of infection, when antibody titers are at undetectable levels [34], and in testing vaccinated patients because previous vaccination does not interfere with the PCR results [35]. Positive PCR results indicate that leptospiral DNA is present in the sample, but negative blood or urine results do not rule out leptospirosis. In fact, leptospiremia is transient, and urinary shedding is delayed after acute infection and can be intermittent. Furthermore, having received an antibiotic treatment recently may affect the detection of leptospiral DNA [1]. For this reason, PCR results should always be interpreted cautiously and in conjunction with MAT results, and they should take into account the clinical context. Furthermore, although its use is highly feasible, routine diagnostic PCR provides no information on the infecting serovar.

Some methods of molecular typing, such as Multilocus Sequence Typing (MLST) [36] and Multiple Loci Variable-number Tandem Repeat Analysis (MLVA) [37], offer interesting epidemiological perspectives by providing a specific and unique barcode for the infecting *Leptospira* through the analysis of specific fragments of particular bacterial loci. Until recently, the MLST technique was only applicable to isolated strains, because it required relatively large amounts of leptospiral DNA, making its direct use on clinical specimens impossible. This was a considerable limitation because culturing leptospires is challenging, time consuming and requires an equipped laboratory. Moreover, the isolation efficiency is very low and is dependent on the *Leptospira* strain and the use of an antimicrobial treatment prior to collection. Fortunately, a fast and specific method for genotyping *Leptospira* DNA directly from biological samples has been developed [38], allowing the rapid identification of the pathogen without strain isolation and providing the opportunity to investigate all the circulating strains, not just those successfully isolated, which represent a small percentage of the spreading strains. Through the assignment of sequence types (STs), MLST permits objective comparisons between strains of *Leptospira* infecting the same host in different geographic regions or different host species within the same geographic region, providing a helpful and powerful tool in investigating the epidemiology of leptospirosis. Therefore, knowledge of regional epidemiology, which can be reliably assessed only by the identification of locally prevalent strains, is necessary for understanding the infection and transmission chains and for maintaining up-to-date vaccination strategies.

The aims of this study were to provide the existent assessment of the genetic diversity in leptospires infecting dogs in Northeast Italy, highlight the need to update vaccine formulations to improve effective preventive measures and reduce the burden of leptospirosis among the canine population.

## 2. Results

### 2.1. Real-Time PCR

Real-time PCR detected pathogenic leptospiral DNA in 264 out of 1631 (16.2%) tested dogs (347 out of 2485 biological samples) (Table 1).

### 2.2. Isolation

In total, 15 strains were successfully isolated, all from urine samples. They were identified at serogroup and serovar level using MAT with polyclonal antisera and monoclonal antibodies (mAbs), respectively (Table 2).

### 2.3. Genotyping Analyzsis

#### 2.3.1. Multilocus Sequence Typing (MLST)

Out of 193 leptospires infecting dogs subjected to a genotyping analysis, the sequence types of 106 were completely defined, 29 were partially defined (4–6 amplifiable loci) and 58 were undefined (0–3 amplifiable loci). Difficulties in amplifying all loci resulted from the low amounts of leptospiral DNA present in the samples. Samples having a partial pattern were considered because they can be still classifiable to unique known STs, which are defined by loci alleles (Table 3).

A total of six known distinct STs were detected, with three STs belonging to *L. interrogans* (ST17, ST198 and ST24), two STs belonging to *L. kirschneri* (ST117 and ST289) and one ST belonging to *L. borgpetersenii* (ST155).

The major portion (68.9%) of *Leptospira* infecting dogs was identified as ST17, which identifies *L. interrogans* serogroup Icterohaemorrhagiae (serovar Icterohaemorrhagiae or Copenhageni). Other portions were identified as follows: 9.6% as ST198, which identifies *L. interrogans* serogroup Australis serovar Australis; 7.4% as ST24, which identifies *L. interrogans* serogroup Australis (serovar Bratislava or Jalna); 5.2% as ST117, which identifies *L. kirschneri* serogroup Pomona serovar Mozdok; 4.4% as ST155, which identifies *L. borgpetersenii* serogroup Sejroe and 3.7% as ST289, which identifies *L. kirschneri* serogroup Pomona.

One sample collected from the Emilia-Romagna region in 2019 showed a new genotype (named as ST17-like) that was characterized by a new allelic combination, similar to that of ST17 (*glmU:* 1, *pntA:* 1, *sucA:* 2, *tpiA:* 2, *pfkB:* 10, *mreA:* 4, *caiB:* 8), but having allele 13 instead of 1 for the *glmU* gene. Its *sucA* gene was not amplifiable and, therefore, its MLST profile was incomplete.

Considering an immunity protection of one year after vaccination [39,40], 12 out of 40 dogs that had knowingly been vaccinated were theoretically protected from the clinical disease while 28 were not protected because they had been vaccinated more than 12 months before sample collections, or never vaccinated (Table 4).

Three regularly vaccinated dogs were clinically affected by *L. interrogans* serogroup Icterohaemorrhagiae (ST17), a serogroup included in all the vaccine formulations. The following related anamnestic and prognostic data were available: one dog was a 5-month old puppy that had regularly received the first dose of the vaccine. The dog was developing symptoms but completely recovered; the second was a hunting dog that received a booster dose two months before but with a fatal outcome because it was treated too late, and the third dog commonly frequented a river near its home and was showing the onset of hyperacute symptoms. However, thanks to therapeutic intervention, it completely recovered.

Three regularly vaccinated dogs were infected by serovars belonging to Pomona and Sejroe serogroups (ST117, ST155 and ST289) that were not included in the vaccine formulations. Four out of six vaccinated dogs infected with serovar Australis ST198 had been vaccinated with bivalent or trivalent vaccines that did not contain antigens of the Australis serogroup and one received a tetravalent vaccine, which contained *L. interrogans* Bratislava within Australis serogroup as the antigen.

Overall, for 39 of 135 dogs, leptospirosis was lethal (Table 4). Among them, 32 (82%) were infected by *Leptospira* ST17, 3 (7.7%) by *Leptospira* ST117 and 1 (2.6%) each by *Leptospira* belonging to ST24, ST155, ST198 and ST289 (Table 4). Interestingly, all the STs were able to cause fatal leptospirosis.

#### 2.3.2. Multiple Loci Variable-Number Tandem Repeat Analysis (MLVA)

The MLVA pattern of 15 isolates and 2 DNAs from biological samples were obtained. The failure to amplify some loci was due to two different causes: the absence of the locus for biological reasons in the species under test (for example the locus Lb4 for *L. interrogans* and the loci 10 and Lb4 for *L. kirschneri*) [37] that was indicated as N (negative) or because of intrinsic causes (i.e., low amount of DNA) that do not permit a successful amplification (in this case we used the term “Not Amplifiable”). Twelve samples (11 isolates and 1 DNA) belonging to *L. interrogans* Icterohaemorrhagiae were genotyped as 2-1-7-N-6 for the loci 4-7-10-Lb4-Lb5, respectively. Additionally, two isolates previously typed as ST198 (*L. interrogans* Australis Australis) were genotyped as 2-10-10-N-5, respectively; two isolates with ST117 (*L. kirschneri* Pomona Mozdok) were genotype as 0-1-N-N-3, respectively, and one DNA of *L. interrogans* Australis Jalna/Bratislava (ST24) was genotyped as 4-10-10-N-Not Amplifiable, respectively. The MLVA patterns did not permit any further discrimination of the strains belonging to the same ST, but they corroborated the results obtained by the MAT and/or MLST analysis.

### 2.4. Sequencing A Tract of the lic12008 Gene on Strains Belonging to ST17

A small tract of the *lic12008* genes in 93 samples (both isolates and DNAs) that belonged to ST17 was sequenced for serovar discrimination. In total, 66 gene tracts were successfully sequenced, with 62 identified as serovar Icterohaemorrhagiae and 4 as serovar Copenhageni (Table 5). No product was obtained owing to the low concentrations of leptospiral DNA from 19 isolates, and 8 DNA samples were insufficient for analysis.

Interestingly, the serovar identifications by *lic12008* sequencing and MAT were in agreement for nine isolates, but not for two isolates that were identified as serovar Copenhageni using the serological method but as serovar Icterohaemorrhagiae using sequencing.

Two dogs infected with *Leptospira* of serovar Icterohaemorrhagiae were regularly vaccinated: one with a tetravalent vaccine and one with a bivalent vaccine, which both contained antigens of serovar Icterohaemorrhagiae. One dog infected with *Leptospira* serogroup Icterohaemorrhagiae, serovar undefined, was regularly vaccinated with a quadrivalent vaccine containing serovar Icterohaemorrhagiae. In total 18 of 62 dogs infected with *Leptospira* serovar Icterohaemorrhagiae were not optimally protected because they had been vaccinated more than 12 months before sample collection or never. For the four dogs infected with serovar Copenhageni, one had never been vaccinated against *Leptospira* infection, while the vaccination states of the three remaining dogs were unavailable.

### 2.5. Data Analysis

#### 2.5.1. Geographical Maps Based on Found Genotypes

**Figure 1:**
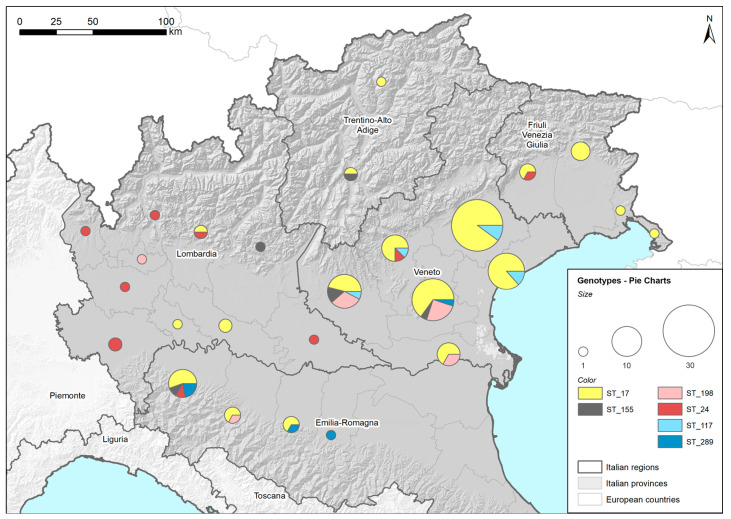
Geographical distribution of Sequence Types (STs). Locations and amounts of genotyped strains, assessed by Multilocus Sequence Typing (MLST), are reported using ST and province in five regions of Northeast Italy (colored in grey) using pie charts. ST17: *L. interrogans* serogroup Icterohaemorrhagiae (serovar Icterohaemorrhagiae or Copenhageni); ST24: *L. interrogans* serogroup Australis (serovar Bratislava or Jalna); ST198: *L. interrogans* serogroup Australis serovar Australis; ST155: *L. borgpetersenii* serogroup Sejroe; ST117: *L. kirschneri* serogroup Pomona serovar Mozdok; ST289: *L. kirschneri* serogroup Pomona.

Figure 1 shows the distribution of the identified STs among the provinces of the five regions of Northeast Italy (Lombardy, Emilia Romagna, Veneto, Trentino Alto Adige and Friuli Venezia Giulia). Both the complete and the partial MLST profiles were reported and placed in a corresponding ST. Two genotyped samples identified as ST17 were excluded from the map because their province could not be determined. The high concentration of genotyped samples in the Veneto region was associated with the large number of samples delivered to the laboratory for *Leptospira*-infection diagnosis owing to the wide involvement of veterinary practitioners in the research, which was less common in the other regions. Despite some variability at the regional level, the STs identified in the tested dogs were found widespread in the investigated area. ST17, the most highly represented ST among the detected genotypes, was present in all five of the regions considered. The limited sampling in some regions may explain why some STs were not detected in all the regions considered.

#### 2.5.2. Phylogenetic Analysis Based on Concatenated MLST Loci

**Figure 2:**
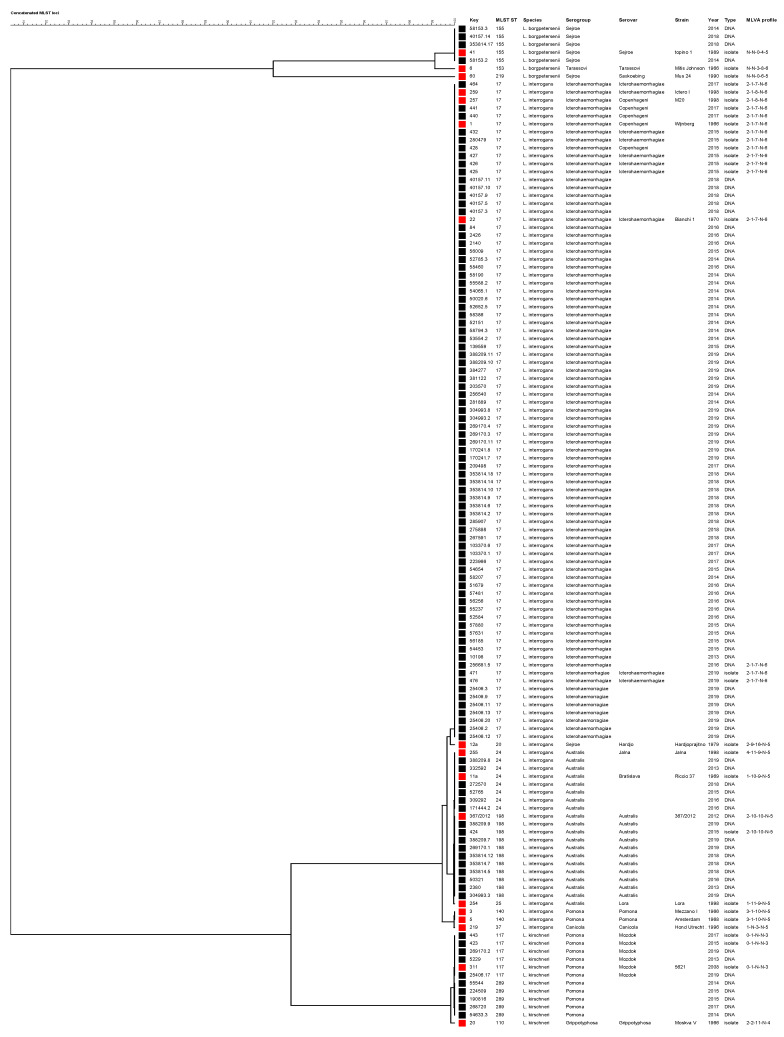
Phylogenetic tree constructed using the 3111-bp concatenated MLST loci. The Unweighted Pair Group Method with Arithmetic mean (UPGMA) was used with a bootstrap analysis based on 1000 replicates. Reference strains are indicated by red squares, while field strains are indicated with black squares. The MLVA pattern of each sample is reported on the right (the order of loci is as follows: VNTR-4, -7, -10, Lb4 and Lb5). N: negative.

A phylogenetic tree was constructed using the sequences of 3111-bp of concatenated MLST loci (Figure 2). Samples genotyped as ST17 clustered with reference strains *L. interrogans* Copenhageni (strains Wijnberg and M20) and Icterohaemorrhagiae (Bianchi 1 and Ictero I). Leptospires genotyped as ST155 clustered with reference strain *L. borgpetersenii* Sejroe strain Topino 1, and those genotyped as ST24 clustered with reference strains *L. interrogans* Jalna Jalna and Bratislava Riccio 37. Leptospires having ST117 clustered with strain *L. kirschneri* Mozdok 5621, while those having ST198 clustered with strain 367/2012, a recently obtained isolated in Istituto Zooprofilattico Sperimentale della Lombardia e dell’Emilia Romagna laboratory from a hedgehog [41], genotyped as *L. interrogans* serovar Australis and shared six out seven loci with ST24. The phylogenetic analysis indicated that ST289 is closely related to *L. kirschneri* serovar Mozdok, as recently reported [42].

#### 2.5.3. Minimum Spanning Tree Based on MLST Data

**Figure 3:**
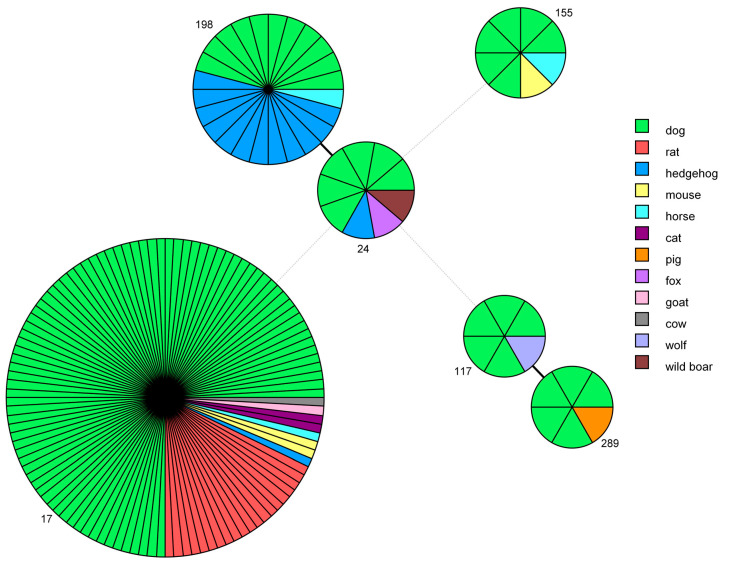
Minimum spanning tree based on sequence types (STs). Each circle represents one specific ST (assigned numbers are located outside the circles), and the number of sectors corresponds to the number of samples having this ST. The tree is color-coded according to host. Solid black lines represent branches between STs that differ at one locus, while dotted light-grey lines represent branches between STs differing at more than four loci.

The genotypes obtained in this study were compared with the STs present in the Istituto Zooprofilattico Sperimentale della Lombardia e dell’Emilia Romagna (IZSLER) database and relative to other host species, using a minimum spanning tree analysis (Figure 3).

ST17, which was very frequently identified in dogs, was also found in 20 rats, 2 mice, 2 cats, 1 hedgehog, 1 horse, 1 goat and 1 cow. ST198 was first identified by the IZSLER laboratory in nine hedgehogs collected in 2012 from a collection center at Modena, Italy [41], and it was subsequently found in three more hedgehogs, one dog and one horse. ST24 was identified also in one hedgehog, one wild boar and one fox. ST289, which was first discovered in a pig in Northern Italy in 2014 [42], is a new genotype that has been identified in five dogs. ST117 was also found in a wolf and ST155 was responsible for the infection of one mouse and one horse.

## 3. Discussion

The absence of available molecular typing data and the complex genomic diversity of strains responsible for canine leptospirosis motivated us to genotype strains from symptomatic dogs in Northeast Italy, sampled between 2013 and 2019, using molecular methods.

Real-time PCR targeting the *lipL32* gene [27] or *rrs* (16S) gene [29] was used to screen the infected dogs and genotyping was conducted to identify the leptospires responsible for the infections.

The isolation of *Leptospira* strains was attempted, but only 15 out of 486 samples were successfully cultured, despite the immediate inoculation of the culture medium with samples. This confirmed that culturing *Leptospira* is difficult [22] and indicates the importance of increasing the tools available for genotyping strains directly from the DNA of biological samples.

The genotyping analysis, which was successfully performed on 15 isolates and 120 leptospiral DNAs, has increased our understanding of the epidemiological status of canine leptospirosis in Northeast Italy. It corroborated the serological evidence of the presence of high frequencies of strains from serogroup Icterohaemorrhagiae (ST17) and Australis (ST24 and ST198), but it also revealed the important roles of other serogroups, such as Pomona (ST117 and ST289) and Sejroe (ST155), as causative agents of the disease. Furthermore, this study revealed the presence of two recent genotypes, ST198 and ST289, which were discovered in hedgehogs and pigs, respectively, in Northern Italy. Thus, the results offer an interesting epidemiological perspective on the circulation of strains among different species and areas.

Among the identified genotypes, ST17, indicating the presence of *L. interrogans* serogroup Icterohaemorrhagiae, was confirmed as the major cause of canine leptospirosis. Previous serological surveys have consistently shown Icterohaemorrhagiae as the most reactive serogroup in dogs suspected of having leptospirosis [13,15,43,44,45,46] and interestingly, the serological profiles of human subjects suspected of having leptospiral infections also indicate that serovars Icterohaemorrhagiae and Copenhageni act as the main causative agents [47,48].

The application of an epidemiological approach, which considered data from the National Reference Centre for Animal Leptospirosis of Istituto Zooprofilattico Sperimentale della Lombardia e dell’Emilia Romagna (IZSLER) related to historical canine samples or belonging to other host species, provided insights into the infection’s transmission chain. Our findings corroborated the hypothesis that dogs are exposed to environmental contamination spread by rodents [49], notably *Rattus* spp., which act as the main reservoir host of Icterohaemorrhagiae/Copenhageni serovars worldwide [50]. Although the inclusion of serogroup Icterohaemorrhagiae in vaccine formulations has led to its decreasing seroprevalence in Italy [46], this serogroup is still a major cause of disease in dogs, owing to the ubiquitous nature of its maintenance host. The dog, in this case, represents a spill-over and can be used as an important sentinel species for human and other animal infections [51,52,53]. Interestingly, the IZSLER database revealed that *Leptospira* ST17 occurs in different hosts (mouse, cat, hedgehog, horse, goat and cow), which indicates its ability to cause infection in a wide range of animal species. The sequencing of the *lic12008* gene, which was recently reported by Santos et al. [54] as useful in discriminating Icterohaemorrhagiae and Copenhageni serovars, allowed us to determine that the major portion (94%) of samples typed as ST17 represented serovar Icterohaemorrhagiae, and only a small portion (6%) represented serovar Copenhageni. The reliability of this molecular test for serovar discrimination is still unclear, and further studies are necessary, given that the serovar determinations of two isolates, as assessed by mAbs and by *lic12008* sequencing, were not in agreement (Table 5).

Three regularly vaccinated dogs were infected by *L. interrogans* serogroup Icterohaemorrhagiae, which is included in all the commercial vaccine formulations, which, however, are commonly able to protect the animal against acute signs but may not prevent infection if the animal is exposed to high bacterial load [15]. An evaluation of the anamnestic and prognostic data of these dogs revealed that they were borderline cases. The puppy, being in a vulnerable developmental period, showed symptoms even after it developed antibodies, but the protection provided by the vaccine may have played a key role in its complete recovery. The hunting dog, which may have been exposed to high bacterial loads that could be responsible for the disease manifestation, started therapy late, resulting in its death. In this case, the delayed treatment of leptospirosis may have allowed the escape of surviving bacteria to the bactericidal effect of antibiotics. The dog that commonly frequented the river and developed acute symptoms with severe hepatonephritis, responded to therapy and completely recovered, probably owing to an active and ready immune response previously stimulated by the vaccine. With the exception of the second case, in which the delay in receiving therapy may have been fatal, vaccination appears to have played a crucial role in protecting dogs from death, given that Icterohaemorrhagiae infections in dogs are often associated with clinical courses culminating in fatal outcomes [15,55,56].

Interestingly, 13 dogs were infected by *L. interrogans* ST198, a new genotype first revealed in hedgehogs of Northern Italy [41] and continually serologically typed as belonging to serovar Australis and serogroup Australis. Very recently, Balboni et al. [57] identified ST198 in a symptomatic dog, providing further evidence of its involvement in canine leptospirosis in Italy. The discovery of this genotype among dogs suggested two important findings: from an epidemiological point of view, a possible direct transmission between species or by indirect infection through a contaminated shared environment, and from a clinical point of view, its presence and ability to cause the clinical presentation of leptospirosis, which can be fatal in Italian dogs.

In this study, a novel genotype, called “ST17-like” because of its similarity to ST17, was discovered in a dog of 2019. Unfortunately, it was unsuitable for submission to BIGSdb because of its incomplete MLST profile. Further confirmation and investigations are required, including the evaluation of its presence in other dogs and hosts in the same area.

More than 7% of the genotyped samples were characterized as ST24 and found worldwide in isolates serologically typed as belonging to serovars Jalna and Bratislava, within serogroup Australis [58]. Unfortunately, because the isolates were unavailable, they were not characterized using mAbs, and consequently, there is no data that can be used to define the specific infective serovars present in the sampled dogs. It is probable that these infections were largely caused by *L. interrogans* serovar Bratislava, because data from the past 30 years indicate the widespread exposure of dogs to this serovar in Europe [11,12,46]. This was confirmed by the isolation of this serovar from urine of infected dogs in Scotland [59] and by its presence among hedgehogs [60], pigs [12] and wild boars [61] in Italy. Bratislava is the serovar most commonly isolated from domestic animals and, for this reason, is most commonly used as the antigen to represent the Australis serogroup in MAT panels. Our study revealed that this strain is responsible for infections in several hosts other than dog, such as hedgehog, wild boar and horse, in agreement with previous serological studies [12,13,61]. Interestingly, in this study, ST24 was also identified in a fox, indicating possible transmission through a contaminated shared environment.

The coverage of the vaccine for the Australis serogroup remains to be evaluated, not only because bivalent vaccines are still widely used but also because new emerging strains, such as *Leptospira* serovar Australis ST198, may represent a significant cause of canine leptospirosis in Italy. Because current tetravalent vaccines contain antigens of Australis Bratislava (ST24), new research is required to evaluate their ability to protect dogs against infection by *Leptospira* Australis ST198.

Strains typed as ST117, indicating the presence of *L. kirschneri* serovar Mozdok, play an important role in causing leptospirosis in dogs. Epidemiological studies have identified this serovar across Europe [17,62,63,64,65], in dogs [19], small rodents [65,66,67], in cattle and pigs [68]. It has been involved in human cases and in canine infections in Cuba [64,66,67,69], making it a relevant risk to public health. Clinically, dogs infected with *Leptospira* belonging to this serogroup experience a severe disease characterized by lethargy, fever, lack of appetite, diffuse hemorrhage and renal and liver failure [70,71]. Serovar Pomona, included in the same serogroup, has emerged in the USA as cause of clinical symptoms in dogs [51], and it has been added to vaccine formulations. In Italy, in 2002, Scanziani and colleagues reported some serological positivity of dogs to Pomona serogroup [11], and recently, Bertelloni found an increasing incidence of Pomona among dogs in the north central area of Italy [13]. In 2010, Ellis [17] had proposed the usefulness of including other serovars, such as Pomona, in the vaccine formulation, but he also believed that additional clinical, cultural and serological studies were needed to support their inclusion. Here, the obtained data agree with previous serological data and support the inclusion of a Pomona serogroup strain, especially one belonging to serovar Mozdok, in dog vaccines in Europe.

Interestingly, five dogs were infected by leptospires genotyped as ST289, a new genotype recently found for the first time by IZSLER laboratory in a pig of 2014, living in Northern Italy [42]. It was typed using core genome MLST as very similar, but not identical, to *L. kirschneri* serovar Mozdok, within serogroup Pomona. The discovery of this genotype in Italian dogs suggested its ability to cause clinical manifestations in dogs and the possible direct transmission among animals or an indirect passage, possibly in a herd context. More studies should be performed to clarify the role of this new strain in causing canine leptospirosis, but our findings support the inclusion of serogroup Pomona in vaccine formulations

In the present study, ST155 was found in six dogs. It characterizes *L. borgpetersenii* serogroup Sejroe, but the serovar status was not deducible from the MLST profile. Nevertheless, the international database BIGSdb [58] reported that isolates typed as ST155 belong to serovars Polonica and Saxkoebing. Using the information from our database, ST155 was compatible with both serovar Sejroe and serovar Polonica, but not with serovar Saxkoebing. In fact, the reference strains serovar Sejroe strain Topino 1, serovar Sejroe strain M24 (Figure 2) and serovar Polonica strain 493 Poland (not reported) were previously typed as ST155, while the reference strain serovar Saxkoebing strain Mus 24 was typed as ST219 (Figure 2). Strains serotyped as Sejroe were previously described as being causative agents of canine leptospirosis worldwide [72,73], and interestingly, this strain was found in an asymptomatic dog in Brazil [74,75]. The minimum spanning tree, color-coded by animal host, revealed the shared presence of these STs among dogs, horses and mice.

Our results indicated that the use of the serovar of the serogroup Sejroe strain in MAT analyses to test local canine populations should be reconsidered. In fact, the MAT panel commonly contains serovar Hardjo (ST152), but the inclusion of serovar Sejroe (ST155) would be more useful for diagnosing canine leptospirosis in our area.

Serogroup Canicola, historically included in commercial vaccine formulations, was not found in this work, and this was in agreement with previous serological studies that describe a decline in the prevalence of this serogroup in European countries, possibly because of the widespread protection resulting from the vaccination of the reservoir host [76], i.e., dog. The risk of stopping vaccinations against host-adapted *Leptospira* in its target host is that its prevalence may rapidly increase once the population’s immunity falls; therefore, the vaccination of dogs against serovar Canicola should continue [17].

Serovar Grippotyphosa is maintained by a number of small rodent species in mainland Europe [77,78,79]. Seroprevalence studies have indicated Grippotyphosa infections in several European countries, including Italy [11], and consequently, it has been included in commercial vaccines. However, in this molecular survey, this strain was not detected among the sampled canine population nor in other species.

The geographical map indicated a widespread distribution of the identified genotypes throughout the considered area, except for ST289, mostly concentrated in Emilia-Romagna region and ST117, found only in Veneto region. Nevertheless, these differences might be attributed to the variability in the numbers of genotyped samples rather than a geographic-dependent diversity in ST circulation. Proper investigations will determine the presence of specific environmental niches for canine leptospirosis or particular risk factors, but the common practice of owners traveling with their pets for recreational activities, such as hiking and hunting, often out of their town or province, should be taken into consideration during risk evaluation. Therefore, when planning dog vaccinations or investigating a suspected clinical case, all of the identified genotypes should be considered epidemiologically relevant.

## 4. Materials and Methods

### 4.1. Sampling, DNA Extraction and Real-Time PCR

In total, 2485 biological samples from 1631 dogs with suspected clinical leptospirosis were tested at IZSLER located in Brescia (Italy) and at Istituto Zooprofilattico Sperimentale delle Venezie (IZSVE) located in Legnaro (Padua, Italy) between January 2013 and December 2019.

Samples were collected by veterinary practitioners in the north central area of Italy (Lombardy and Emilia-Romagna regions) and in Northeast Italy (Veneto, Friuli Venezia Giulia and Trentino- Alto Adige regions) during routine diagnostic activities, owing to a systematic passive survey funded by the Italian Ministry of Health and coordinated by the IZSVE, involving many veterinary practitioners of the region. When available, data on the vaccination states and clinical follow-ups of dogs were also recorded.

DNA extractions and real-time PCR targeting the *lipL32* gene were carried out at IZSLER as previously described by Bertasio et al. [42], while at IZSVE, different DNA extraction methods and kits were used in accordance with the variety of samples. A commercially available High Pure PCR Template Preparation kit (Roche Diagnostics, Mannheim, Germany) was used, in accordance with the manufacturer’s instructions, to extract 2 mL of urine or 1 cm^3^ tissue homogenate. From 1 mL of EDTA-treated blood, DNA was extracted using a commercially available QIAamp DNA Mini kit (Qiagen, Hilden, Germany), in accordance with the manufacturer’s instructions. In the case of poor-cellular matrices, as in urine and blood samples, 20 µg of a poly-A carrier (Roche, Diagnostics, Mannheim, Germany) was added to each sample to increase the recovery efficiency of nucleic acids. All the DNA extraction preparations included a negative control (water). All the DNAs extracted from biological samples were subjected to a TaqMan-based real-time PCR assay targeting an 87-bp fragment that corresponded to a portion of the gene encoding the 16S rDNA [29].

The PCR was performed in a 25 µL final volume, containing 3 µL of extracted DNA, 12. 5 µL of 2× Master Mix TaqMan Universal 2× (Thermo Fisher Scientific, Waltham, MA, USA), 300 nM of each primer and 100 nM of a 5′ FAM–3′-TAMRA probe. All the amplification assays included a negative control (water), a negative bacterial genomic control (DNA of *Leptospira biflexa* serovar Patoc) and a positive control (DNA of *L. interrogans* serovar Icterohaemorrhagiae), and each sample was tested in duplicate. The assay was performed on a 7900HT Fast Real-time PCR System (Thermo Fisher Scientific) with the following thermal conditions: a hot-start step at 50 °C for 2 min, a holding step at 95 °C for 10 min and 45 cycles of 95 °C for 15 s and 60 °C for 60 s. Samples with C_t_ < 38 were considered positive. Samples having C_t_ values within the 38–40 range were considered doubtful, whereas samples having no FAM fluorescence signal or with C_t_ ≥ 40 were considered negative.

The two methods were validated using reference materials and their performance monitored by a regular participation to proficiency testing programs organized by the National Reference Laboratory, with an agreement index (K Cohen) equal to 1.

### 4.2. Isolation and Serological Typing of Isolates

In total, 486 samples from 449 clinically affected dogs were chosen to attempt the isolation of the causal agent as previously described [42]. The specific matrices of these samples were 416 urines, 35 kidney and 11 livers, while for 24 samples, the data were unavailable.

The preliminary serogroup assignment was carried out using MAT with polyclonal serogroup-specific antibodies, as previously described [42]. The serovars were classified using MAT with different panels of mAbs for Icterohaemorrhagiae, Pomona and Australis serogroups. The mAbs used were the following: five mAbs (F20 C4, F52 C1, F70 C24, F70 C26 and F82 C1) for the Icterohaemorrhagiae serogroup [80], five mAbs (F43 C9, F46 C9, F48 C6, F58 C1 and F61 C7) for the Pomona serogroup [81] and six mAbs (F81 C3, F81 C5, F81 C6, F81 C8, F90 C6 and F90 C8) for the Australis serogroup [82]. These mAbs and the polyclonal anti-sera were previously purchased from the OIE Leptospirosis Reference Centre, Royal Tropical Institute (KIT), (Amsterdam, The Netherlands), and the protocol was in accordance with the standard serological methods used in this reference laboratory. To interpret the results, we referred to the expected maximum dilution titers provided by the Leptospirosis Reference Centre of KIT.

### 4.3. Genotyping

#### 4.3.1. MLST

Until 2015, MLST analyses were applied only to DNA samples with C_t_ ≤ 35 as assessed by real-time PCR, and starting from 2016, the use of MLST was extended to all DNA samples. If more than one biological matrix was available for the same dog, the analysis was attempted on all the samples to maximize the probability of success. In total, 223 samples (15 isolates and 208 leptospiral DNAs) belonging to 193 dogs were submitted for MLST analysis (Table 6).

The analysis was performed using the 7-loci scheme proposed by Boonsilp in 2013 [36], which is based on the housekeeping genes *glmU*, *pntA*, *sucA*, *tpiA*, *pfkB*, *mreA* and *caiB*, as previously described [42]. Nucleotide sequences were assembled using the SeqMan module of the Lasergene sequencing analysis software package (DNASTAR Inc., Madison, WI, USA) or using Bionumerics software ver. 7.6 (Applied Math, Biomerieux, Sint-Martens-Latem, Belgium). For allelic number and ST identification, assembled and trimmed sequences were queried against the BIGSdb available on the *Leptospira* MLST website (https://pubmlst.org/leptospira/) sited at the University of Oxford [58] or by an automated analysis using Bionumerics. Comparisons between the STs found and those present in BIGSdb as reference isolates were used to deduce the species of the *Leptospira* being tested. To perform comparisons among historical serological studies (where serovars and serogroups were defined) and genotyping data (where species and genomic profiles were defined), we chose to assign to each identified ST a classification at the serogroup and serovar levels obtained from BIGSdb, knowing that this information was deduced and did not result from active serological typing.

#### 4.3.2. MLVA

A VNTR analysis was performed on 21 samples (15 isolates and 6 DNAs). Five discriminatory loci (VNTR-4, -7, -10, Lb4 and Lb5) described by Salaün and colleagues in 2006 [37] were considered, as previously described [42].

### 4.4. Sequencing of a Tract of the lic12008 Gene

A tract of the *lic12008* gene of samples genotyped as ST17 (characterized as *L. interrogans* serogroup Icterohaemorrhagiae) was sequenced. The tract was recently found to be involved in the genetic distinction between serovars Icterohaemorrhagiae and Copenhageni [54], which are indistinguishable by MLST and MLVA. The analysis determines the presence of a single base insertion of a thymine nucleotide within a poly-thymine tract (9-bp long) in the *lic12008* of all *L. interrogans* serovar Icterohaemorrhagiae isolates but not in Copenhageni strains. We used gene-specific primers (forward 5′-TAGGTTGGCACGAAGGTTCT-3′ and reverse 5′-CTTAAACTTTCCACTTTCCGGA-3′) to amplify a short 163-bp genomic tract containing the described discriminatory INDEL.

The PCR amplification was performed using a KAPA2G Robust HotStart PCR kit (Kapabiosystems Resnova, Rome, Italy) in a 25 µL total volume containing 0.4 µM each primer and 5 µL of DNA. The thermal conditions were as follows: 1 cycle at 95 °C for 7 min, 45 cycles at 95 °C for 30 s, 53 °C for 30 s and 72 °C for 30 s, followed by a final elongation at 72 °C for 5 min. For the sequencing procedure, we followed the same protocol used for the MLST analysis. Nucleotide sequences were assembled using the SeqMan module of the Lasergene sequencing analysis software package (DNASTAR) and were aligned with the sequences of Copenhageni and an Icterohaemorrhagiae reference strains to permit a comparison and to define the serovar of the tested *Leptospira* strain.

### 4.5. Analysis of Data

#### 4.5.1. Geographical Map

The provinces in which the dogs were living were recorded to construct a map that displayed the distributions of the identified genotypes. The map was produced using the commercial software ESRI^TM^ArcMap 10.5.1. The spatial layer containing the provinces was joined (using province ID attribute) with the sample data. Specific style roles were applied to generate different pie charts based on the presence of different STs. The size of each chart represents the total number of genotyped samples per province. Each chart reports the results (as percentages) per ST (denoted by different colors).

#### 4.5.2. Phylogenetic Analysis of Concatenated MLST Loci

The concatenated loci assessed by MLST on our samples were compared with those of reference strains purchased from the Leptospirosis Reference Centre of KIT using the UPGMA method with a bootstrap of 1000 replicates by Bionumerics ver. 7.6 (Applied Maths, Sint-Martens-Latem, Belgium). The resulting tree reported sample details, such as the identification number, the species, the serological classification (real for isolates and presumptive for DNA samples) and the MLVA pattern.

#### 4.5.3. Minimum Spanning Tree based on STs

An analysis of the characteristics of alleles assessed by MLST was performed by the creation of a minimum spanning tree using Bionumerics software ver. 7.6 (Applied Maths). The analysis was applied only to samples having a complete MLST profile, and to perform an epidemiological evaluation, we also considered data for some samples already present in the IZSLER database but external to the period included in this paper (six isolated strains from dogs: 2, 1, 1, 1 and 1 from 1992, 1995, 1996, 1999 and 2007, respectively) or belonging to other hosts species (20 from rat, 3 from mouse, 2 from cat, 14 from hedgehog, 3 from horse, 1 from cow, 1 from goat, 1 from pig, 1 from wolf and 1 from wild boar). The IZSLER database is an internal database file that includes updated information on the genotyping and serological typing of isolated strains preserved in liquid nitrogen, on field samples from various animal species collected from 1989 and on reference strains purchased from by the Leptospirosis Reference Centre of KIT. Furthermore, starting in 2016, data from the molecular typing of leptospiral DNAs extracted directly from biological samples of a series of animal hosts have been added.

## 5. Conclusions

To the best of our knowledge this is the first study that describes the genetic diversity of pathogenic *Leptospira* in dogs in Italy, and it has increased our understanding of the related epidemiological situation among the Italian canine population. No area of specific risk was revealed in Northeast Italy, and the widespread presence of already known infecting serogroups, like Icterohaemorrhagiae and Australis, was confirmed. Interestingly, it highlighted the circulation of infecting strains belonging to other serogroups, such as Pomona and Sejroe, that are not included in the commercial vaccines currently available in Italy. The strains of these two serogroups were responsible for clinical leptospirosis, which was sometimes fatal, in dogs. Furthermore, new strains recently observed in other hosts in Italy, like ST198 from hedgehogs and ST289 from pigs, may play important roles in causing canine leptospirosis in Italy.

## Figures and Tables

**Table 1:** Results of real-time PCR performed on canine biological samples.

	Urine	Blood	Kidney	Liver	Lung	Bladder	Spleen	Organ(s)	Not Defined	Total
**No. of Positives/Total**	162/1095	66/665	75/392	23/115	7/33	2/9	0/6	12/148	0/22	347/2485
**Percentage of Positives**	14.8%	9.9%	18.6%	20.0%	21.2%	22.2%	0.0%	8.1%	0.0%	14.0%

**Table 2:** Isolated samples and their serological typing assessed by the Microscopic Agglutination Test.

No. of Isolates	Species	Serogroup	Serovar
8	*L. interrogans*	Icterohaemorrhagiae	Icterohaemorrhagiae
3	*L. interrogans*	Icterohaemorrhagiae	Copenhageni
2	*L. kirschneri*	Pomona	Mozdok
2	*L. interrogans*	Australis	Australis

**Table 3:** Multilocus Sequence Typing (MLST)-based genotyping using the seven-loci scheme developed by Boonsilp et al. [36].

Genotyped Samples	ST 17	ST 198	ST 24	ST 117	ST 155	ST 289	ST 17 Like	Total
**No. of DNA**	65 (+17)	8 (+3)	6 (+4)	3 (+2)	4 (+2)	5	(1)	91 (+29)
**No. of Isolates**	11	2		2				15
**Total**	76 (+17)	10 (+3)	6 (+4)	5 (+2)	4 (+2)	5	(1)	135
**%**	68.9%	9.6%	7.4%	5.2%	4.4%	3.7%	0.7%	100.0%

The numbers of samples having complete MLST patterns are reported. Numbers in parentheses indicate the additional samples having a partial pattern. ST: sequence type.

**Table 4:** Vaccination and vital states of sampled dogs and the STs of the infecting *Leptospira.*

ST of Infecting *Leptospira*	Vaccination Status			Vital Status			Total
	Regular	Not Regular	Unknown	Alive	Died	Unknown	
17	3	23	65	5	32	56	91
198	6	1	6	4	1	8	13
24		1	9		1	9	10
117	1	1	5	1	3	3	7
155	1	2	3		1	5	6
289	1		4		1	4	5
17 Like			1			1	1
Total	12	28	95	10	39	86	135

Regular: if the last vaccination occurred less than 12 months before sampling; Not regular: if the vaccination occurred more than 12 months before sampling or never.

**Table 5:** The *lic12008* gene sequencing of strains previously typed as ST17.

No.	Type	Species	Sequence Type	Serovar Definition	
				By *lic12008* Sequencing	By MAT
8	isolate	*L. interrogans*	17	Icterohaemorrhagiae	Icterohaemorrhagiae
2	Isolate	*L. interrogans*	17	Icterohaemorrhagiae	Copenhageni
1	Isolate	*L. interrogans*	17	Copenhageni	Copenhageni
52	DNA	*L. interrogans*	17	Icterohaemorrhagiae	N.A.
3	DNA	*L. interrogans*	17	Copenhageni	N.A.

Serovar identification by *lic12008* sequencing and MAT are compared. N.A.: not applicable.

**Table 6:** Samples submitted for MLST analysis: details of their matrices.

Matrix	No. of DNA Extracted from Biological Samples	No. of Isolated Samples	Total
Urine	101	15	120
Blood	45		45
Kidney	34		34
Lung	5		5
Organ(s)	6		6
Liver	4		4
Bladder	3		3
Not defined	10		10
Total	208	15	223
